# SOX9 and TNFAIP3 dysregulation in HCV-associated HCC after DAA therapy: insights into post-viral oncogenic memory

**DOI:** 10.1186/s13027-026-00735-w

**Published:** 2026-02-19

**Authors:** Rehab I. Moustafa, Sally Farouk, Hassan Elsayed, Hend I. Shousha, Maha M. Elbrashy, Ahmed M. Gabr, Ahmed A. Yousif, Ahmed Ramadan, Ayman Yosry, Ashraf O. Abdelaziz, Amr Abdelaal, Noha G. Bader El Din

**Affiliations:** 1 Microbial Biotechnology Department, Biotechnology Research Institute, National Research Centre, Dokki, Cairo, 12622 Egypt; 2 Endemic medicine and Hepato-gastroenterology Department, Faculty of Medicine, Cairo University, Cairo, Egypt; 3 Biochemistry Department, Biotechnology Research Institute, National Research Centre, Dokki, Cairo, 12622 Egypt; 4 Department of Surgery, Faculty of Medicine, Modern University for Technology and Information, Cairo, Egypt; 5 Hepato-Gastroenterology and Infectious Disease Department, Ahmed Maher Teaching Hospital, Cairo, 11613 Egypt; 6 Department of Surgery, Faculty of Medicine, Ain Shams University, Cairo, Egypt

**Keywords:** HCV-induced HCC, SVR, PBMCs, SOX9, TNFAIP3, FOSL2, Immune infiltration

## Abstract

**Background:**

Hepatitis C virus (HCV) is a leading cause of chronic liver disease and hepatocellular carcinoma (HCC). Despite the high efficacy of direct-acting antivirals (DAAs) in eradicating HCV, HCC may still develop after sustained virological response (SVR), suggesting that HCV may leave behind lasting epigenetic and immunological alterations that sustain oncogenic risk.

**Objectives:**

This study aimed to investigate the expression profiles of SOX9, TNFAIP3, and FOSL2 in peripheral blood mononuclear cells (PBMCs) and liver tissues from HCV-related HCC patients and to explore, through in silico analyses, their molecular and immunological roles in HCV-driven hepatocarcinogenesis.

**Methodology:**

Gene expression was quantified in PBMCs and liver tissues using RT-qPCR. Receiver operating characteristic (ROC) curve analyses assessed diagnostic potential. In silico analyses evaluated protein-protein interactions, gene-gene networks, epigenetic modifications, and correlations with immune cell infiltration and immunomodulatory molecules using publicly available datasets.

**Results:**

SOX9, TNFAIP3, and FOSL2 were identified as interconnected regulators within NF-κB and TGF-β pathways, enriched in inflammatory and infection-related processes, and epigenetically modulated via promoter hypermethylation and histone remodeling. Their expression strongly correlated with macrophages, T cells, dendritic cells, and key immune modulators. RT-qPCR validation confirmed overexpression of SOX9 and TNFAIP3 in PBMCs and liver tissues from HCV-HCC patients, with PBMC levels closely reflecting tissue expression, and ROC analyses highlighted their potential as non-invasive biomarkers.

**Conclusions:**

SOX9 and TNFAIP3 emerge as key mediators linking persistent epigenetic alterations with immune remodeling in HCV-related HCC, and as potential non-invasive biomarkers for evaluation of HCC risk and post-DAA surveillance.

**Clinical trial number:**

Not applicable.

**Supplementary Information:**

The online version contains supplementary material available at 10.1186/s13027-026-00735-w.

## Introduction

Hepatitis C virus (HCV) remains a major global health challenge, driving chronic liver injury, cirrhosis, and hepatocellular carcinoma (HCC), which ranks as the third leading cause of cancer-associated deaths worldwide [1]. The development of direct-acting antivirals (DAAs) has markedly advanced HCV therapy, achieving sustained viral clearance in most individuals. However, the extent to which DAA therapy restores hepatic and immune homeostasis remains controversial.

Several studies report persistent innate and adaptive immune abnormalities, including peripheral NK cells, monocytes, and CD4+, CD8 + T cells [2–6], as well as persistent risk of HCC after viral clearance [7–9]. Abnormal serum lipids [10], sustained hepatic inflammation [11], and persistent epigenetic modifications have also been observed following treatment [12, 13]. Generally, these findings suggest that HCV-induced epigenetic dysregulation, including aberrant chromatin remodeling, DNA methylation, and microRNA regulation, may persist as an “oncogenic memory” after viral eradication [13–16].

The intrinsic regenerative capacity of the liver also represents a key contributor to hepatocarcinogenesis after sustained virological response (SVR). Hepatocyte-driven regeneration requires extensive chromatin remodeling and activation of proliferative pathways, which may predispose to malignant transformation [17, 18]. Chronic HCV infection further reinforces this vulnerability through direct viral effects, core and NS5A proteins modulate host signaling, impair DNA repair, generate oxidative stress, and induce persistent epigenetic alterations, including histone modifications and DNA methylation changes that may persist after viral clearance. These epigenetic imprints can sustain aberrant expression of oncogenic drivers and repression of tumor suppressors, thereby maintaining a pro-tumorigenic transcriptional program, contributing to residual HCC risk after SVR [13, 14, 16]. Parallel disruptions in innate and adaptive immunity, including impaired NK-cell function, altered T-cell responses, and attenuation of type I/III interferon signaling after rapid viral clearance, may transiently weaken tumor immunosurveillance and contribute to residual HCC risk despite elimination of viremia [2, 3, 19–21].

Persistent epigenetic dysregulation is closely intertwined with immune remodeling in the liver. Tumor-infiltrating immune cells, including macrophages and lymphocytes, reinforce oncogenic signaling through cytokine secretion and crosstalk with hepatocytes, thereby driving angiogenesis, immune evasion, and tumor progression. Peripheral blood mononuclear cells (PBMCs), which constitute the first line of immune defense, comprising monocytes and lymphocytes, undergo phenotypic and transcriptional reprogramming in response to tumor-derived signals. In HCV-induced HCC, heightened immune infiltration and sustained cytokine release may coordinately upregulate immune-regulatory and oncogenic transcriptional programs both locally within the liver and systemically in circulating PBMCs. Consequently, PBMC gene expression reflects tumor immune dynamics and serves as a non-invasive surrogate for assessing immune evasion, host–tumor interactions, and disease prognosis, overcoming the limitations of invasive liver biopsy procedures [22–26]. Such persistent epigenetic and immunological reprogramming may sustain a pro-tumorigenic microenvironment and contribute to HCC development through complex host–immune interactions. Consequently, individuals with advanced liver fibrosis retain HCC risk, underscoring the need for continued post-treatment surveillance [27–29].

Several immune-regulatory genes are jointly influenced by inflammatory signaling and epigenetic reprogramming, notably SOX9, TNFAIP3, and FOSL2, which play critical roles at the intersection of oncogenesis and immune modulation. SOX9, a key transcription factor involved in liver development, contributes to HCC progression through epigenetic regulation and oncogenic signaling. Promoter hypomethylation and persistent H3K27ac histone modifications following DAA-mediated HCV cure sustain its overexpression, driving tumor-initiating cell properties and promoting hepatocarcinogenesis [13, 30, 31].

TNFAIP3 (A20), a ubiquitin-editing enzyme and NF-κB negative regulator, displays a context-dependent role in HCC [32–34]. Normally anti-inflammatory and tumor-suppressive, its elevated expression may promote immune evasion and resistance to apoptosis through PI3K/Akt and Bcl-2 pathways [35, 36]. Furthermore, epigenetic reprogramming after HCV infection, including increased H3K9 acetylation at the TNFAIP3 locus, may sustain its overexpression and immune-regulatory effects even after viral eradication [37].

FOSL2, (Fra-2), an AP-1 transcription complex component, mediates pro-inflammatory and fibrogenic signaling to regulate macrophage activation and hepatic remodeling during chronic liver injury [38, 39]. FOSL2 also plays a pivotal role in HCC pathogenesis through epigenetic regulation, influencing epithelial–mesenchymal transition (EMT), immune evasion, and tumor heterogeneity [40, 41].

In HCV-related HCC, persistent inflammatory and epigenetic perturbations may cooperatively upregulate these genes in both hepatic tissue and circulating immune cells.

However, no previous Egyptian studies have examined their expression in HCV-infected patients following DAA therapy. Therefore, this study aimed to investigate, for the first time, the expression patterns of SOX9, TNFAIP3, and FOSL2 in PBMCs and liver tissues from Egyptian patients with HCV-related HCC, to elucidate their association with immune infiltration and systemic immune modulation. We analyzed PBMC gene expression in 220 subjects (170 patients and 50 controls) and validated these findings in 60 liver tissue samples. The study cohort included HCC patients infected with HCV genotype 4, both DAA-treated and untreated, along with untreated HCV patients without HCC and HCC cases of other etiologies. By exploring the interplay between HCV-induced epigenetic reprogramming and oncogenic pathways, this study seeks to uncover potential new biomarkers for assessing HCC risk, diagnosis, and targeted therapy, ultimately contributing to mitigating the substantial HCC burden in Egypt and advancing the field of precision hepatology.

## Materials and methods

### Study design

This study integrates observational molecular profiling of clinical samples with in silico analyses of HCC liver transcriptomic datasets. In silico analyses are used to evaluate the dysregulation of SOX9, TNFAIP3, and FOSL2 in HCC and to assess their involvement in infection- and inflammation-related pathways, as well as their associations with immune cell composition in HCC tissues. To evaluate their potential as minimally invasive biomarkers, a two-stage cross-sectional design was employed, comprising RT–qPCR analysis of gene-expression patterns across defined clinical groups in PBMCs from 170 patients, followed by validation in a paired liver tissue cohort of 60 matched specimens from a subset of the same individuals, enabling direct within-subject comparison.

### In silico analyses

The UALCAN Database Portal (https://ualcan.path.uab.edu) was utilized to compare gene expression differences of studied genes between tumor and adjacent normal tissues in various tumors. Hierarchical clustering analysis of SOX9, TNFAIP3, and FOSL2 was conducted using the University of California, Santa Cruz (UCSC) Cancer Genomics Browser (http://genome-cancer.ucsc.edu). To visualize the correlation of the expression of the studied genes with the level of immune infiltration in HCC, we used the gene module of Timer portal (https://cistrome.shinyapps.io/timer/), while the TISIDB database (http://cis.hku.hk/TISIDB/search.php) was used to correlate each gene with immune stimulators in HCC. The network construction module of the PINA.v3 portal was employed to analyze protein-protein interactions (PPI) among the investigated genes, specifying TCGA-LIHC as the cancer type. After that, the list of proteins of the PINA network was inserted into the Enrichr portal (https://maayanlab.cloud/Enrichr/enrich#) for visualizing PINA network as Gene Ontology (GO) and Kyoto Encyclopedia of Genes and Genomes (KEGG) Pathway enrichment plots. The Gene expression profiling interactive analysis (GEPIA2) database (http://gepia2.cancer-pku.cn/#index) was utilized to assess the correlation between histone remodelers and target genes based on TCGA-LIHC datasets. The resulting data were visualized as dot-line correlation plots using SRplot [42]. Additionally, the cBioPortal TCGA-LIHC database (https://www.cbioportal.org/) was utilized to assess the correlation between mRNA expression levels of each gene and their corresponding DNA methylation patterns.

### Subjects and study groups

Patients were recruited from multiple medical centers, including the Air Force Specialized Hospital (Cairo, Egypt), Ahmed Maher Teaching Hospital (Cairo, Egypt), HCC Multidisciplinary clinic, Kasr El Ainy Hospital, and the Center for Hepatic Fibrosis (Center of Excellence, STDF-5274), Faculty of Medicine, Cairo University (Cairo, Egypt). All participants provided informed consent in line with the Declaration of Helsinki, and the study was approved by the Medical Research Ethics Committee (FWA 00014747) of the National Research Centre (NRC), Cairo, Egypt (registration number 20–113). All the clinical and pathological data were documented by qualified medical professionals following the study’s inclusion and exclusion criteria.

The PBMC cohort included five groups: 1.Post-SVR–HCC: patients with previous chronic HCV infection who achieved sustained virological response following DAA therapy and subsequently developed HCC, included to assess gene-expression changes associated with post-SVR hepatocarcinogenesis (*n* = 65). Patients with chronic hepatitis C were treated according to the Egyptian national guidelines for the prevailing genotype 4 using a 12-week sofosbuvir/daclatasvir, regimen, with adjunctive ribavirin administered based on clinical judgement, predominantly in cases of compensated cirrhosis or prior treatment exposure [43, 44]. SVR was defined as undetectable serum HCV RNA (≤ 15 IU/mL) at least 12 weeks after treatment completion. 2. DAA-naïve HCV–HCC: patients diagnosed with HCV infection during diagnostic evaluation for HCC who had not received antiviral therapy (*n* = 35). These patients typically presented with advanced or end-stage tumors that precluded DAA treatment, representing HCV-related HCC in the absence of DAA-associated immunologic modulation. 3.CHC-no HCC: patients with active chronic HCV infection, no evidence of HCC at the time of sampling, and no prior exposure to DAA therapy (*n* = 35). This group served to distinguish transcriptional and immune alterations related to chronic viral infection from those associated with malignant transformation. 4.Non-HCV- HCC: patients with HCC of non-HCV etiology (e.g., NASH or alcoholic liver disease)(*n* = 35), included to assess whether identified signatures were specific to HCV-associated hepatocarcinogenesis or shared across diverse etiologies. 5. Healthy Controls, individuals without known liver disease or HCV infection (*n* = 50), serving as a baseline reference for gene-expression normalization.

### HCC surveillance, diagnosis, and staging

Patients achieving sustained virologic response (SVR), particularly those with advanced cirrhosis, underwent standardized hepatocellular carcinoma (HCC) surveillance in accordance with international guidelines. Surveillance included clinical assessment, serum alpha-fetoprotein (AFP) testing, and abdominal ultrasonography at 6-month intervals. Suspected HCC was confirmed by dynamic contrast-enhanced magnetic resonance imaging (MRI) and/or computed tomography (CT) according to American Association for the Study of Liver Diseases (AASLD) and European Association for the Study of the Liver (EASL) criteria.

Tumor staging across the HCC groups was performed using the Barcelona Clinic Liver Cancer (BCLC) classification, a prognostic framework that integrates tumor burden (size, multifocality, vascular invasion, and metastasis), liver function assessed by the Child–Pugh score, and patient performance status.

Paired liver tissue samples were available from a subset of participants (*n* = 60), including specimens from SVR–HCC (*n* = 15), HCV–HCC (*n* = 15), and Non-HCV–HCC (*n* = 15) patients, as well as normal liver tissues from healthy controls (*n* = 15), thereby enabling direct comparison of gene-expression patterns in liver tissue and corresponding PBMCs from the same individuals.

Exclusion criteria included the presence of any significant comorbidity such as cardiac, respiratory, or renal failure; malignancies other than HCC; uncontrolled systemic infections; and psychiatric disorders.

### Total RNA extraction from study biospecimens

For PBMCs, total RNA was isolated using the QIAamp^®^ RNA Blood Mini Kit (#52304; Qiagen). RNA concentration and purity from both tissue and PBMC samples were measured using a Thermo Scientific NanoDrop 2000c Spectrophotometer (RRID: SCR_020309), ensuring suitability for further molecular applications.

Regarding the liver tissue specimens, they were promptly immersed in RNAlater Tissue Reagent (Qiagen, Hilden, Germany) to preserve RNA integrity and stored at − 70 °C until processing. Samples were then lysed in Buffer RLT and homogenized using a Qiagen TissueLyser LT (Qiagen, Hilden, Germany) to ensure complete disruption. Subsequently, total RNA was extracted using the AllPrep DNA/RNA/Protein Mini Kit (Qiagen), in accordance with the manufacturer’s instructions.

### Quantitation of SOX9, TNFA1P3, FOSL2 genes relative expression via qRT-PCR

Equal quantities of total RNA were reverse transcribed into cDNA using the QuantiTect Reverse Transcription Kit (#205311; Qiagen, Hilden, Germany), following the manufacturer’s protocol to eliminate genomic DNA contamination. Briefly, RNA samples were first treated with gDNA Wipeout Buffer and RNase-free water, followed by incubation at 45 °C for 8 min. The reverse transcription reaction was then performed by adding Quantiscript Reverse Transcriptase, RT buffer, and RT primer mix, and incubated sequentially at 25 °C for 3 min, 45 °C for 20 min, and 85 °C for 5 min to inactivate the enzymes.

Afterward, Real-time PCR was performed using the QuantiNova SYBR Green PCR Kit (#208052; Qiagen) to quantify relative mRNA expression. Each reaction included synthesized cDNA, 2×SYBR Green Master Mix, commercially designed QuantiTect SYBR primers #249900; Qiagen), and RNase-free water. Amplification was carried out on a Rotor-Gene PCR cycler (Qiagen, Hilden, Germany) under the following thermal cycling conditions: initial enzyme activation at 95 °C for 2 min, followed by 40 cycles of denaturation at 95 °C for 5 s, annealing at 60 °C for 10 s, and extension at 70 °C for 30 s. Gene expression levels were normalized to the housekeeping gene β-actin, and the relative fold changes were calculated using the ΔΔCT method.

### Biostatistical analysis

All statistical analyses were performed using GraphPad Prism version 9. Data were presented as mean ± standard deviation (SD). Group comparisons were conducted using the Mann–Whitney U test for two-group comparisons, while Kruskal-Wallis test was applied when comparing more than two groups. Spearman’s rank correlation test was used to assess the strength and direction of associations between variables. The diagnostic potentials of the target genes SOX9, TNFAIP3, and FOSL2 were evaluated using receiver operating characteristic (ROC) curve analysis, with the area under the curve (AUC) used to determine discriminative ability and identify optimal cutoff values. A p-value < 0.05 was considered statistically significant throughout the study.

## Results

### Expression of SOX9, TNFAIP3, and FOSL2 across TCGA tumor and normal samples

**Fig. 1:**
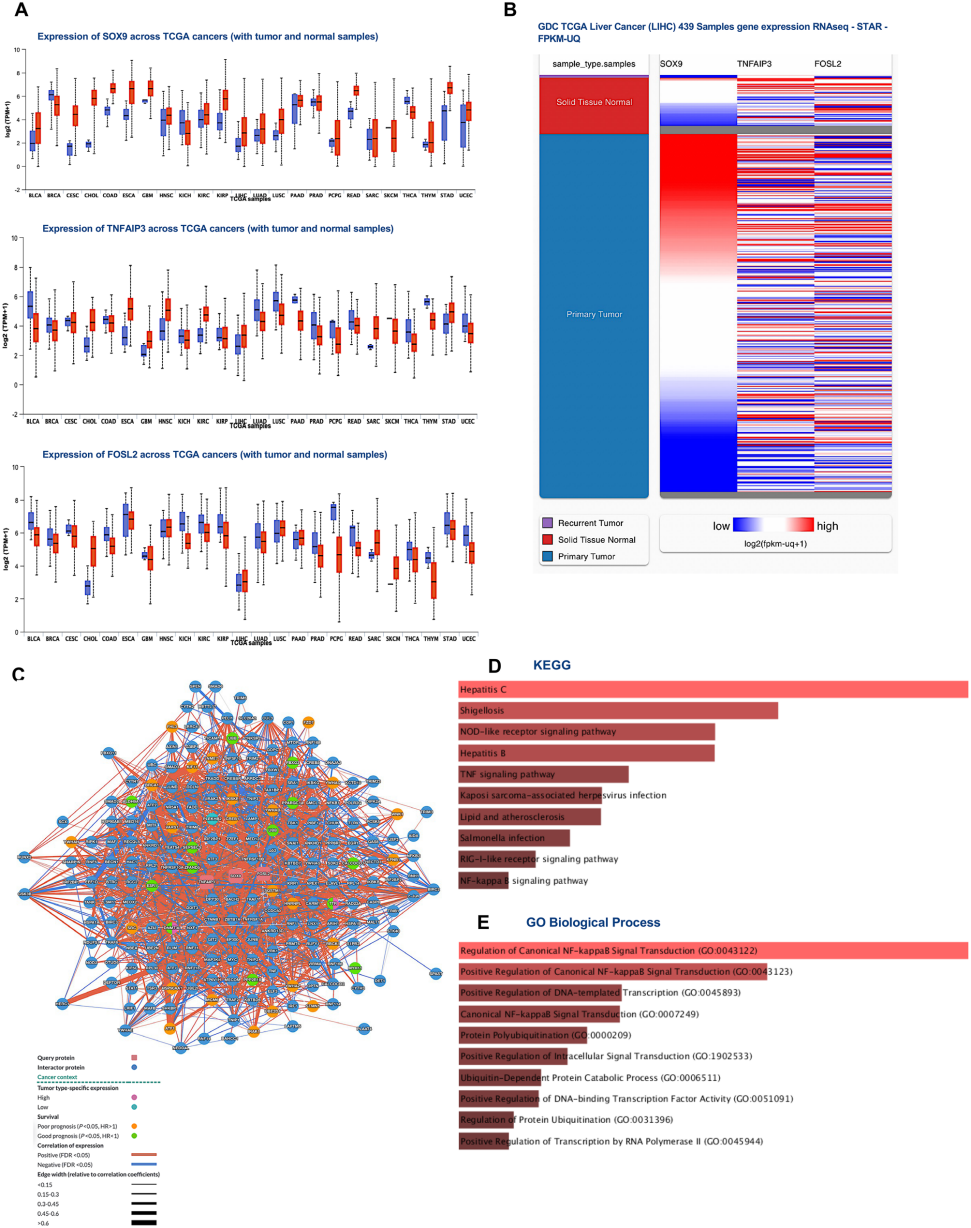
**A**) Expression of SOX9, TNFAIP3, and FOSL2 across TCGA Tumor and Normal Samples. Boxplots show the expression levels (log2 TPM + 1) of SOX9, TNFAIP3, and FOSL2 across multiple TCGA cancer types, comparing tumor (red) and matched normal (blue) samples. Each panel represents one gene: top left, SOX9; middle, TNFAIP3; bottom, FOSL2. Cancer types are indexed on the x-axis, labeled with TCGA abbreviations (e.g., BRCA, LIHC). Boxes represent interquartile ranges; whiskers indicate variability outside quartiles. **B**) Hierarchical clustering of SOX9, TNFAIP3, and FOSL2 genes was built with the help of the University of California Santa Cruz genomics browser. The upregulated genes are denoted by red, and the downregulated genes are denoted by blue. **C**) PPI Network and Enrichment Analysis for SOX9, TNFAIP3, and FOSL2 in HCC. **D**) KEGG pathway enrichment results for PPI network genes via Enrichr. **E**) Gene Ontology (GO) Biological Process enrichment of the network genes

The expression patterns of the candidate genes across different cancer types were investigated by analyzing TCGA transcriptomic data through the UALCAN database. The results revealed that SOX9 exhibited elevated transcript levels in multiple tumor types, most prominently in liver hepatocellular carcinoma (LIHC), bladder carcinoma (BLCA), cholangiocarcinoma (CHOL), and esophageal carcinoma (ESCA), while showing minimal tumor vs. normal differences in kidney renal clear cell carcinoma (KIRC) and head and neck squamous cell carcinoma (HNSC). TNFAIP3 was generally upregulated in tumors compared to normal tissues in cancers such as ESCA and LIHC but showed reduced or unchanged expression in kidney chromophobe (KICH) and stomach adenocarcinoma (STAD). Similarly, FOSL2 expression was significantly increased in several malignancies, including LIHC, lung squamous cell carcinoma (LUSC), and CHOL, but downregulated or unchanged in others, such as KICH and prostate adenocarcinoma (PRAD). Collectively, these data highlight distinct, cancer type–specific expression patterns, with all three genes showing marked deregulation across the TCGA spectrum, most notably elevated in LIHC, suggesting their potential involvement in hepatocarcinogenesis (Fig. 1A).

### Hierarchical clustering analysis of SOX9, TNFAIP3, and FOSL2 expression in TCGA-LIHC

To further characterize the expression landscape of SOX9, TNFAIP3, and FOSL2 within hepatocellular carcinoma, hierarchical clustering analysis was performed using gene expression data from the TCGA Liver Cancer (LIHC cohort). The analysis revealed distinct expression patterns among SOX9, TNFAIP3, and FOSL2 (Fig. 1B). SOX9 showed markedly elevated and consistent expression in primary tumor samples compared with solid tissue normal controls, where expression levels remained uniformly low, supporting its potential role as a central oncogenic driver in hepatocellular carcinoma. In contrast, TNFAIP3 and FOSL2 exhibited heterogeneous expression profiles across tumor samples, with variable clusters of high and low expression. This heterogeneity indicates that the expression of TNFAIP3 and FOSL2 may be influenced by tumor subtype–specific characteristics or microenvironmental factors, unlike the more consistent activation observed for SOX9.

### Interaction network analyses and functional enrichment analysis of SOX9, TNFAIP3, and FOSL2 in HCC

Given the distinct expression patterns observed among our genes of interest, network and functional enrichment analyses were performed to investigate their possible molecular interactions and biological pathway involvement. The PINA portal PPI analysis of SOX9, TNFAIP3, and FOSL2 in HCC revealed a highly interconnected and complex network of interacting proteins (Fig. 1C). The network exhibited cancer- specific changes in expression, with both positive and negative correlation among the proteins. The presence of strongly connected hub proteins suggests their central role in mediating key biological processes relevant to HCC progression.

Functional enrichment analysis using the Enrichr portal demonstrated that the PPI-associated genes are predominantly enriched in infection- and immune-related KEGG pathways, including Hepatitis C, Hepatitis B, Shigellosis, NOD-like receptor signaling, and TNF signaling (Fig. 1D), highlighting their relevance to infection-driven liver inflammation and carcinogenesis. The biological process enrichment in Gene Ontology (GO) revealed that the associated genes are predominantly involved in regulatory and signaling functions, particularly within the canonical NF-κB signaling pathway. The most significantly enriched terms included positive regulation of NF-κB signal transduction, regulation of protein ubiquitination, and activation of transcription factor activity via RNA polymerase II (Fig. 1E). Together, these results indicate that the PPI-associated genes, including SOX9, TNFAIP3, and FOSL2 are integral components of intracellular signaling and transcriptional regulation networks. By participating in key inflammatory and protein modification pathways, they are likely to contribute to tumor progression, immune modulation, and maintenance of cellular homeostasis in hepatocellular carcinoma.

### Association of SOX9, TNFAIP3 and FOSL2 gene expression and immune cell infiltration in HCC

To further elucidate the immunological relevance of our candidate genes, we analyzed their association with immune cell infiltration in HCC using publicly available datasets. Since PBMC-derived gene expression may reflect systemic immune modulation, examining tumor immune infiltration patterns provides complementary insight into the tumor–immune interface. This analysis helps determine whether the dysregulated expression of SOX9, FOSL2, and TNFAIP3 corresponds to specific immune cell populations within the tumor microenvironment, thereby linking peripheral immune signatures to local immune dynamics in HCC.

**Fig. 2:**
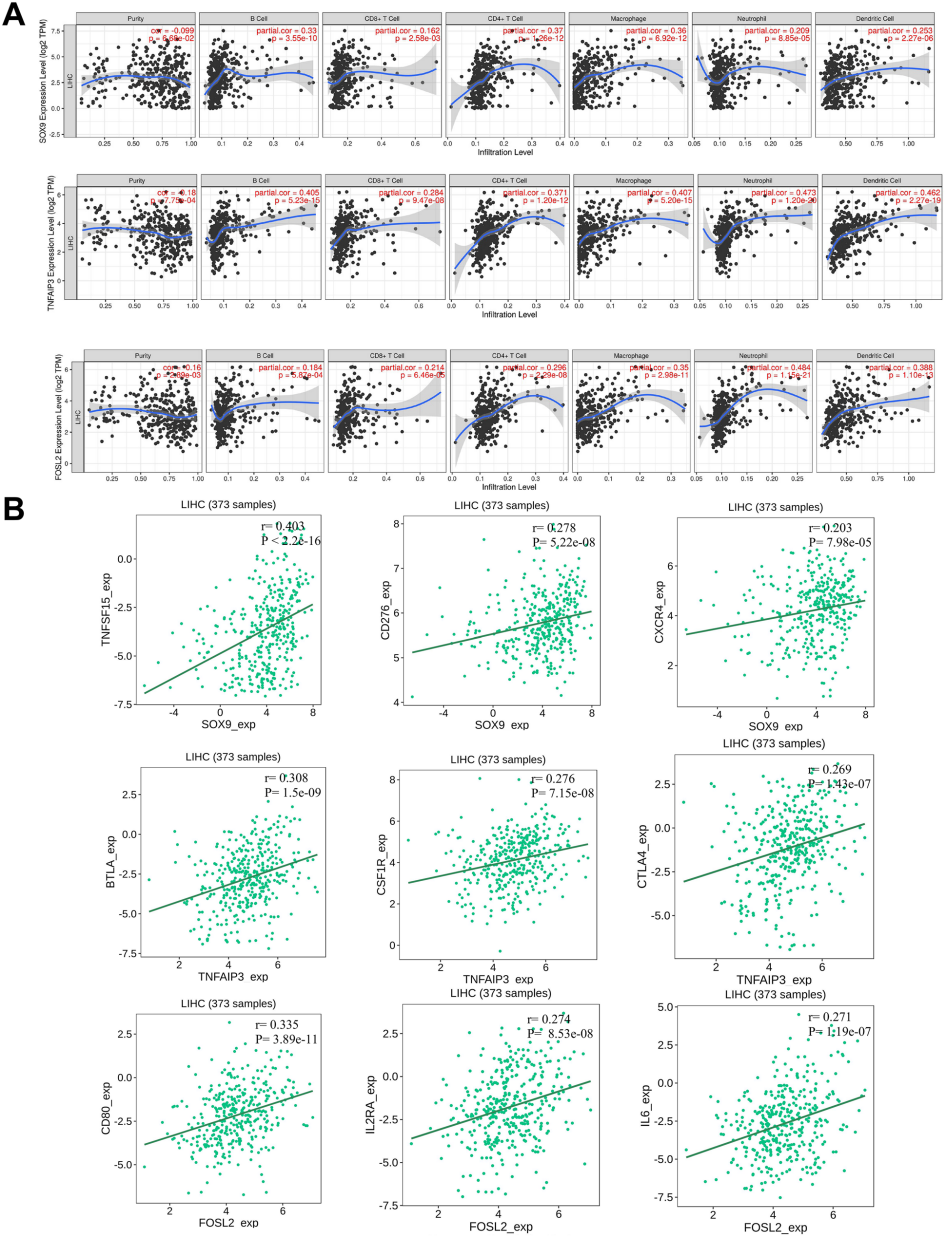
**A**) Correlations Between SOX9, TNFAIP3 and FOSL2 Expression and Immune Cell Infiltration in HCC. Scatterplots showing the association between mRNA expression (log2 TPM, y-axis) of SOX9 (top row), TNFAIP3 (middle row), and FOSL2 (bottom row) infiltration levels of different immune cells (x-axis) in HCC. Each subplot represents a specific cell type: tumor purity, B cell, CD8 + T cell, CD4 + T cell, macrophage, neutrophil, and dendritic cell. Each graph indicates the partial Spearman correlation coefficient and p-value (in red) for each gene-immune cell pair. **B**) Correlations between gene expression of SOX9, TNFAIP3 and FOSL2 with immune-stimulatory genes in HCC samples. Scatter plots showing statistically significant positive correlations between the expression levels of SOX9, TNFAIP3 and FOSL2 genes and multiple immune stimulatory genes in 373 LIHC samples. Correlations of SOX9 (top row), TNFAIP3 (middle row), and FOSL2 (bottom row) with corresponding immunostimulatory genes are shown, with linear regression trend lines overlaid. The correlation coefficient (r) and p-value for each comparison are indicated in the top right corner of each plot

Expression analyses of SOX9 revealed significant positive correlations with multiple immune cell populations, including B cell (partial cor = 0.33), CD4 + T cells (0.37), macrophages (0.36), neutrophils (0.209), and dendritic cells (0.253). These findings support the involvement of SOX9 in modulating immune cell recruitment and shaping the tumor–immune microenvironment in liver and other cancers.​​ TNFAIP3 exhibited even stronger correlations, particularly with B cells (partial cor = 0.405), CD4 + T cell (0.371), macrophages (0.407), neutrophils (0.473), and dendritic cells (0.462). Consistent with its established role as a regulator of NF-κB signaling and immune responses, the elevated TNFAIP3 expression may indicate enhanced activation of inflammatory and immune signaling pathways within HCC. FOSL2 also displayed robust associations with immune infiltration in HCC, especially CD8 + T cells (partial cor = 0.214), CD4 + T cells (0.296), macrophages (0.35), neutrophils (0.484), and dendritic cells (0.388), indicating a potential role in recruiting cytotoxic and antigen-presenting cells to the tumor microenvironment.​​ Collectively, these results indicate that elevated expression of SOX9, TNFAIP3 and FOSL2 is associated with increased immune infiltration, particularly of macrophages, neutrophils, and T cells, highlighting their potential immunomodulatory roles in HCC (Fig. 2A).

Complementary in silico analysis using the TISIDB database further supported these associations, revealing significant positive correlations between gene expression and several immunostimulatory molecules. SOX9 expression showed robust correlations with TNFSF15, CD276, and CXCR4. On the other hand, TNFAIP3 expression was significantly associated with BTLA, CSF1R and CTLA4, while FOSL2 expression was significantly correlated with the expression of CD80, IL2RA and IL6 in 373 HCC samples.​ These associations underscore their potential involvement in immune activation, chemotaxis, and costimulatory signaling, highlighting their coordinated influence on immune dynamics within the HCC microenvironment (Fig. 2B).

### Epigenetic modulation of SOX9, TNFAIP3 and FOSL2 expression in HCV-induced hepatocarcinogenesis

To investigate the molecular mechanisms underlying the dysregulation of SOX9, TNFAIP3 and FOSL2 in HCC we conducted a comprehensive in silico analysis focusing on epigenetic regulation driven by HCV infection. Specifically, we explored how DNA methylation and histone modifications may influence the expression of these genes, contributing to HCC progression, as previously reported [13, 45].

**Fig. 3:**
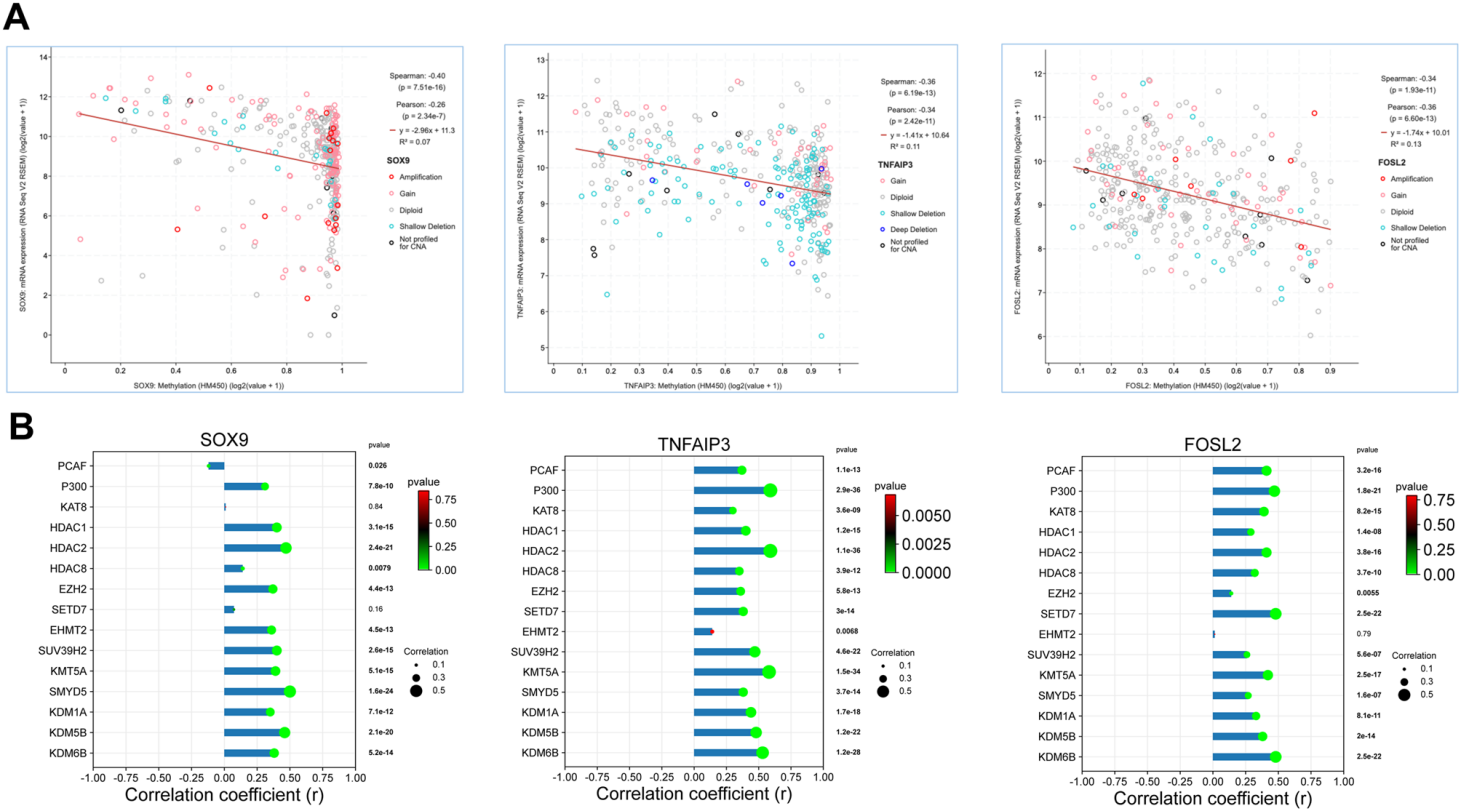
In silico analysis of epigenetic regulatory mechanisms governing SOX9, TNFAIP3 and FOSL2 Expression in HCC. **A**) Correlation plots showing the association between DNA promoter methylation (beta-values) and mRNA expression for the three genes. **B**) Correlation dot line plots depicting the correlation between the mRNA expression of studied genes and the expression of selected histone-modifying enzymes in the TCGA-LIHC dataset. Green dots indicate a statistically significant correlation (*p* < 0.05). The size of the dot is proportional to the absolute correlation value

First, we examined the association between promoter methylation and mRNA expression levels (Fig. 3A). A strong inverse correlation (*p* < 0.05) was observed for all three genes, indicating that hypermethylation plays a major role in their transcriptional repression.

To further explore the epigenetic landscape, we assessed the correlation between gene expression and a panel of histone-modifying enzymes (Fig. 3B). Our findings revealed significant associations with various chromatin remodelers, including histone methyltransferases (EHMT2, EZH2, KMT5A, SETD7, SMYD5, SUV39H2), and histone demethylases (KDM6B, KDM5B KDM1A ), histone deacetylases (HDAC1, HDAC2, HDAC8), histone acetyl transferases (KAT8, P300, PCAF). Although most correlations were significant, some (e.g., FOSL2–EHMT2, SOX9–KAT8, and SOX9–SETD7) were not statistically robust.

Collectively, these findings suggest that coordinated epigenetic mechanisms, including promoter hypermethylation and histone remodeling, contribute to the transcriptional regulation of SOX9, TNFAIP3 and FOSL2 in HCC, supporting their potential roles in HCV-related oncogenesis [46].

Following the in silico analyses, experimental validation was conducted to assess the expression of SOX9, TNFAIP3, and FOSL2 in clinical samples. Gene expression was quantified in PBMCs and liver tissues from HCV-related HCC patients and control groups to validate the computational predictions in a clinically relevant cohort.

### Clinical and pathological data of the studied groups

The clinicopathological characteristics of all study groups are displayed in Table 1. The mean age of the control group was 48 years, while the mean ages of the patient groups were 55 years for Post-SVR-HCC, 59 years for DAA-naïve HCV-HCC, 56 years for CHC-no HCC, and 46 years for non-HCV-HCC. The control group has an equal distribution of males and females, whereas male predominance was observed across all patient groups.

Generally, the control group maintained normal biochemical parameter levels. Only significant differences were found between the control and patient groups in the serum levels of total bilirubin and alpha-fetoprotein (AFP), with *P* = 0.0001 for both markers. In our Post-SVR–HCC cohort, the median time from end of treatment to HCC diagnosis was 22 months (IQR: 14–38 months).

**Table 1:** Clinical and pathological data of the studied groups

	Control (*n* = 50) mean ± SD	Patients *n* = 170				*P* value
		**Post-SVR-HCC***n* = 65	**DAA-naïve HCV-HCC***n* = 35	**CHC-no HCC***n* = 35	**Non-HCV-HCC***n* = 35	
Age (years)	48 ± 2.12	55 ± 1.9	59 ± 1.34	56 ± 2.62	46 ± 1.7	0.4616
Gender M/F	25 (50%) / 25(50%)	48 (73.8%) / 17(26.2%)	29 (82.8%) / 6(11.2%)	26 (74.2%) / 9(25.8%)	21 (60%) / 14(400%)	
Hb (g/dL)	13.2 ± 0.421	11.9 ± 0.667	12.3 ± 0.376	10.8 ± 0.578	12.5 ± 0.348	0.9994
ALT (IU/L)	18 ± 1.22	52 ± 2.175	64 ± 4.097	98 ± 4.749	78 ± 5.43	0.7780
AST (IU/L)	26 ± 1.682	65 ± 2.478	87 ± 6.916	102 ± 6.978	79 ± 3.664	0.8563
T BiL (mg/dL)	0.41 ± 0.106	2.1 ± 0.496	1.30 ± 0.302	1.4 ± 0.184	0.8 ± 0.078	< 0.0001*
ALB (g/dL)	4.7 ± 0.0517	2.8 ± 0.1001	1.8 ± 0.211	1.9 ± 0.201	3.1 ± 0.604	0.9327
PLT (×10^3^/ µL)	295 ± 11.97	125.0 ± 15.11	150.0 ± 13.73	158.0 ± 10.14	222.5 ± 10.34	0.9173
WBCs (×10^3^/µL)	6.9 ± 0.4743	3.9 ± 0.4231	5.3 ± 0.4272	6.0 ± 0.3215	6.05 ± 0.412	0.9143
AFP (ng/mL)	0.51 ± 0.121	1.23 ± 0.1392	1.48 ± 0.2472	1.8 ± 0.2262	450 ± 10.89	< 0.0001*
Ascites Yes	-	49 (75%)	23 (65.7%)	21 (60%)	19 (54.2%)	
Splenomegaly Yes	-	38(58.4%)	19 (54.28%)	20 (57.14%)	13(37.14%)	
BCLC tumor staging (% [*n*/total])						
A B C	- - -	11 (16.9%) 29 (44.4%) 25 (38.4%)	8 (22.8%) 21 (60%) 6 (17.14%)	- - -	10 (28.5%) 19 (54.2%) 6 (17.14%)	

### Screening the relative expression of SOX9, TNFA1P3, FOSL2 among studied groups

**Fig. 4:**
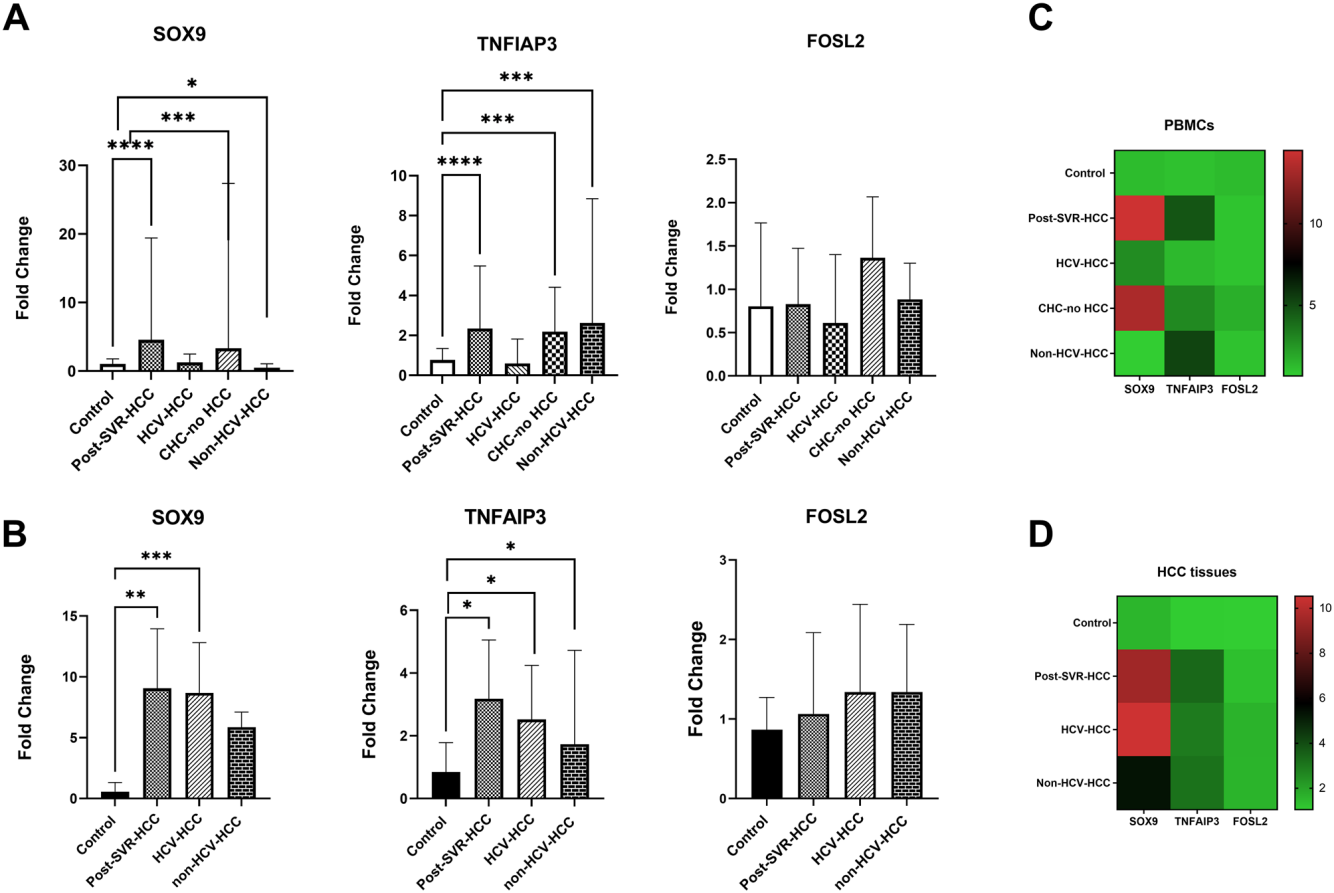
**A**) The differential mRNA expression levels of SOX9, TNFAIP3, and FOSL2 in PBMCs in different study groups compared to healthy controls, respectively. **B**) The differential mRNA expression levels of SOX9, TNFAIP3, and FOSL2 in HCC tissue samples from the study groups compared to healthy controls. Asterisks indicate statistically significant differences between the compared groups (***P < 0.0001, ***P < 0.001, **P < 0.01, *P < 0.05). **C** and **D**) Heatmap illustrating the mean fold change in expression levels of SOX9, TNFAIP3, and FOSL2 across the studied groups in both PBMCs and HCC tissue samples, respectively. The heatmap highlights distinct gene expression patterns associated with each group, providing a visual overview of differential expression in peripheral blood and liver tissue

Differential gene expression in PBMCs among the various groups is illustrated in Fig. 4A. The expression of SOX9 in PBMCs was significantly overexpressed in Post-SVR-HCC patients (mean fold change = 14.48, *P* = 0.0001) and in HCV-non-HCC patients (13.31, *P* = 0.0006), while its expression in DAA-naïve HCV-HCC was 2.757, *P* = 0.7420. Conversely, SOX9 was significantly downregulated in non-HCV-HCC patients (0.6795, *P* = 0.0193), suggesting a distinct expression pattern dependent on both HCV infection and treatment status. Similarly, TNFAIP3 mRNA was markedly upregulated in Post-SVR-HCC, CHC-no HCC, and non-HCV-HCC patients (fold changes of 4.703, 2.964, and 5.282, respectively; all *P* < 0.0001), while showing no significant change in the DAA-naïve HCV-HCC group (1.322, *P* = 0.5855). In contrast, FOSL2 expression in PBMCs showed non-significant variations across all studied groups, indicating limited diagnostic relevance (Supplementary Table S1).

On the other hand, the distinct expression patterns of the studied genes in liver tissues across the different groups are shown in Fig. 4B. Both SOX9 and TNFAIP3 were significantly overexpressed in liver tissue across all HCC groups compared to controls (Supplementary Table S1). SOX9 expression was highest in Post-SVR-HCC (10.53, *P* = 0.0003), followed by DAA-naïve HCV-HCC (9.485, *P* = 0.0013), and non-HCV-HCC (5.337, *P* = 0.0005), reinforcing its association with HCV and DAA treatment. Likewise, TNFAIP3 showed consistent upregulation in these groups (fold changes ~ 2.9–3.4; all *P* < 0.05), supporting its role in liver tumor biology. In contrast, FOSL2 mRNA expression in liver tissues exhibited non-significant changes in all groups, further confirming its limited diagnostic or biological contribution in HCC. To provide a comprehensive overview of gene expression patterns across all study groups, we generated a heat map of the normalized expression levels of SOX9, TNFAIP3, and FOSL2 in both PBMCs and liver tissues (Fig. 4C and D). The heat map revealed distinct expression signatures corresponding to each patient group. Collectively, these findings highlight SOX9 and TNFAIP3 as putative molecular markers of HCC, particularly in the context of HCV infection and DAA treatment, whereas FOSL2 shows limited and inconsistent diagnostic value in both blood and liver tissues.

### Correlation between the expression of studied genes in HCC tissues and PBMCs

**Fig. 5:**
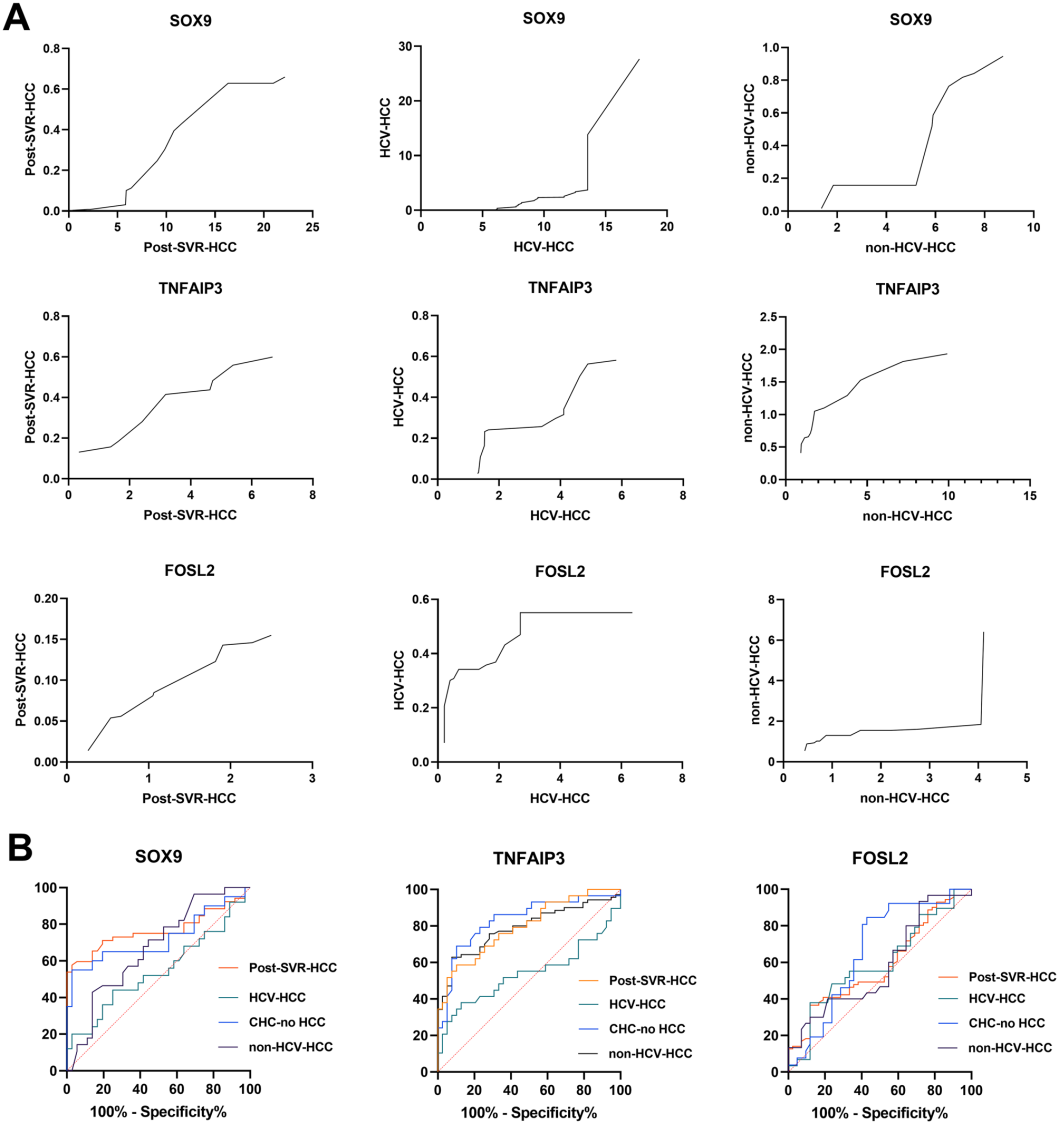
**A**) Correlation analysis between PBMCs and HCC tissue samples. The analysis revealed a strong positive association in the expression levels of the three selected genes (SOX9, TNFAIP3, and FOSL2) across peripheral blood and liver tissue. **B**) ROC curves illustrating the diagnostic performance of SOX9, TNFAIP3, and FOSL2 relative expression levels in PBMCs. The curves demonstrate each gene’s ability to discriminate between HCC-related patient groups and healthy controls

The expression levels of the studied genes exhibited a strong positive correlation between PBMCs and their corresponding liver tissues across all patient groups (Supplementary Table S2). Specifically, SOX9 expression showed significant correlations with *r* = 0.9654, 0.7514, and 0.8977 (*P* < 0.0001) in the post SVR-HCC group, DAA-naïve HCV-HCC group, and non HCV-associated HCC patient group, respectively. Similarly, TNFAIP3 expression demonstrated robust correlations with *r* = 0.9779, 0.9097, and 0.9355 (*P* = 0.0001) across the same groups. FOSL2 also showed positive correlations with *r* = 0.9823 (*P* = 0.0001), 0.7946 (*P* = 0.0012), and 0.7333 (*P* = 0.0019) in post-SVR-HCC, DAA-naïve HCV-HCC, and non-HCV-HCC patients, respectively. These results emphasize the concordance of gene expression profiles between tissue and PBMCs in HCC, as shown in Fig. 5A.

### Diagnostic capacity of SOX9, TNFA1P3, FOSL2 across studied groups

The ROC curve analysis revealed differential diagnostic performance of the studied genes across different patient groups (Fig. 5B). SOX9 expression demonstrated strong diagnostic value, particularly in post-SVR-HCC patients, with a highly significant AUC of 0.92 and a specificity of 97.22% (*P* < 0.0001), indicating its potential utility in this clinical context. Similarly, SOX9 showed a good diagnostic profile in chronic HCV patients (AUC = 0.84, *P* = 0.0008). In contrast, its performance was poor in DAA-naïve HCV-HCC (AUC = 0.52) and only moderate in non-HCV-HCC cases (AUC = 0.67) (Supplementary Table S3). Similarly, TNFAIP3 demonstrated robust diagnostic accuracy in multiple groups. It achieved a high AUC in post-SVR-HCC patients (0.85) and CHC-no HCC (0.83), both with strong statistical significance (*P* < 0.0001), as well as in non-HCV-HCC (AUC = 0.79, *P* < 0.0001). These findings support TNFAIP3 as a broadly applicable biomarker across diverse HCC-related conditions. However, similar to SOX9, its performance was not significant in DAA-naïve HCV-HCC (AUC = 0.54), indicating that the inflammatory or treatment status may influence its expression dynamics. In contrast, FOSL2 consistently showed weak and mostly non-significant diagnostic power across all groups. Its performance was non-significant in all HCC groups (post-SVR-HCC, DAA-naïve HCV-HCC, and non-HCV-HCC; AUC < 0.60), with the exception of a modest, significant association in the chronic HCV cohort (AUC = 0.66, *P* = 0.0224).

## Discussion

HCV remains highly prevalent worldwide and is a major cause of irreversible liver scarring [47]. Viral activation of hepatic stellate cells (HSCs) triggers collagen deposition, which promotes the progression from liver fibrosis to cirrhosis and ultimately hepatocellular carcinoma [48–50]. Extensive research has deepened our understanding of HCV pathogenesis, viral entry, and associated inflammatory mechanisms, which paved the way for the development of direct-acting antivirals (DAAs) [51, 52]. Although early-stage HCV infection can now be effectively treated and cured, the fibrosis developed during the infection often remains irreversible. Although the treatment with DAAs has produced high SVR rates in HCV-infected patients, their impact on host epigenetic modifications remains incompletely understood and continues to be an active area of investigation. To that end, our *in silico * epigenetic correlation analyses revealed strong inverse associations between promoter methylation and mRNA expression of SOX9, TNFAIP3, and FOSL2, highlighting DNA methylation as a key regulatory mechanism in HCC. Moreover, significant correlations with histone-modifying enzymes suggest coordinated histone remodeling that may sustain aberrant transcriptional activity. These findings align with previous evidence of persistent HCV-induced epigenetic reprogramming, which can preserve oncogenic and immune-altering transcriptional states even after viral clearance with DAA therapy [13, 37]. Together, these findings strengthen the evidence that these genes are pivotal epigenetically dysregulated drivers of hepatocarcinogenesis.

Therefore, in the current study, we evaluated the dysregulations of SOX9, TNFAIP3, and FOSL2 genes in PBMCs and liver tissue samples from four distinct groups of Egyptian patients, including post-SVR-HCC, DAA-naïve HCV-related HCC patients, chronic HCV-infected individuals without HCC, HCC patients of other etiologies, along with healthy controls. In the SVR–HCC cohort, the median interval of 22 months between treatment completion and HCC diagnosis is consistent with previous reports indicating that residual HCC risk is greatest within the first 2–3 years after SVR, particularly in patients with advanced fibrosis or cirrhosis [53–55]. Our results revealed significant persistent upregulation of both SOX9 and TNFAIP3 genes, while FOSL2 showed only slight, non-significant variation relative to controls in PBMCs and HCC liver tissues across the studied patient groups.

PBMC-based biomarkers require validation against the tissue of disease origin. The consistent expression of SOX9 and TNFAIP3 in both PBMCs and matched liver tissue shows that circulating immune-cell transcriptional patterns genuinely reflect oncogenic and immune-regulatory processes occurring within the liver. This supports the feasibility of PBMC-based surveillance in post-DAA settings.

Within this validated liver–PBMC framework, SOX9 emerged as the most strongly dysregulated gene in post-SVR HCC. SOX9 expression in PBMCs was significantly upregulated in post-SVR-HCC patients (mean fold change 14.48, *P* = 0.0001) compared to both DAA-naïve HCV-HCC (mean fold change 2.757) and non-HCV-related HCC samples (mean fold change 0.6795). This elevated expression pattern was further validated in corresponding liver tissue specimens. The current findings suggest a potential association between active HCV infection and mediated tumor progression. The transcription factor SOX9 plays a key role in oncogenesis, fibrosis, and liver regeneration. Our results support previous studies that documented the vital role of SOX9 in biliary differentiation and EMT in cirrhotic livers and HCV-related hepatocellular carcinoma [56–58]. Although DAAs effectively eradicate HCV and lead to SVR, recent studies showed persistent elevation of SOX9 expression post-treatment, which may reflect the ongoing activation of regenerative pathways or unresolved fibrotic remodeling [13]. The consistent SOX9 upregulation observed in our study aligns with the findings of Wu et al. [59], who also reported the increase of SOX9 expression in HCV-cured patients. These results further support the concept of “epigenetic scarring”, which remains after viral clearance, potentially contributing to an elevated risk of HCC in DAA-treated individuals.

Similarly, our results in post-SVR-HCC patients following DAA therapy showed upregulation of TNFAIP3 with a statistically significant difference compared to the control group (mean fold change 4.703, *P* = 0.0001). Additionally, analysis of liver tissue consistently demonstrated a similar upregulation of TNFAIP3 expression. TNFAIP3, which encodes A20 protein, inhibits the NF-κB signaling and inflammatory responses. Its upregulation post-DAA therapy may mitigate persistent inflammation, fibrosis, and HCC progression [33, 60, 61]. Furthermore, TNFAIP3 overexpression in colorectal cancer (CRC) inhibited cell proliferation, invasion, and migration [62]. Despite the tumor-suppressive function of TNFAIP3, it has also been reported to play a paradoxical oncogenic role in certain cancers. For example, its upregulation in B-cell lymphomas has been associated with resistance to apoptosis and cancer progression [63].

In HCC patients, TNFAIP3 expression levels can either be overexpressed or downregulated, depending on the cellular environment. In our study, the higher TNFAIP3 expression in both post-SVR-HCC and chronic HCV patients confirms the results of Huang et al., who reported high TNFAIP3 expression levels in cancerous tissues [64]. Previous studies have also linked TNFAIP3 upregulation to enhanced cancer cell survival, which leads to poor prognosis in breast cancer and esophageal cancer patients [65]. Notably, TNFAIP3 was reported to inhibit apoptosis in HCC cell lines, contributing to tumor persistence [66, 67]. Our results support the recent study of Perez et al., 2019 [45] who documented a significant elevation in TNFAIP3 expression level in liver tissues from DAAs-treated HCV patients with advanced fibrosis. These findings suggest that sustained TNFAIP3 overexpression in cured HCV patients can promote hepatocyte survival and facilitate malignant transformation

Conversely, FOSL2 expression did not show statistically significant differential expression in our dataset. A small numerical reduction was observed in PBMCs (mean fold change 0.97), alongside a modest numerical increase in paired liver tissues from post-SVR-HCC. While these non-significant trends do not support definitive biological conclusions, the opposing direction of change in blood versus tissue compartments presents an interesting observation. Future studies with larger cohorts are warranted to determine if DAA therapy is associated with compartment-specific modulation of FOSL2 expression that could be relevant to its known roles in oncogenesis and fibrosis. Notably, FOSL2 protein is known to contribute to tumor growth, cell division, and fibrosis. Interestingly, our results diverge from several previous studies, which reported that FOSL2 mediates EMT, metastasis, and progression of different cancer types [68, 69]. Moreover, the upregulation of FOSL2 has been linked to the fibrogenic activation in the liver stellate cells [70] and suppression of apoptosis in ovarian cancer cells [71]. Consequently, should future studies confirm a downregulation following DAA therapy, it might reflect a favorable change in antifibrotic gene expression, indicating partial resolution of the fibrotic process. Hence, further experimental studies are required to evaluate this potential mechanism.

However, this also accords with our in silico transcriptomic analysis from the TCGA cohort, which revealed heterogeneous expression patterns of TNFAIP3 and FOSL2, while SOX9 exhibited strong and uniform SOX9 upregulation in tumor tissues. This might explain the non-significant variation in FOSL2 expression in the present HCC cohort, suggesting subtype- or microenvironment-dependent regulation. Moreover, protein–protein interaction and enrichment analyses revealed that these genes are integrated into networks enriched for infection- and inflammation-related pathways, such as Hepatitis B/C, NOD-like receptor, and TNF signaling. Functional annotation highlighted their involvement in NF-κB activation, protein ubiquitination, and transcriptional regulation, supporting roles in inflammatory and oncogenic signaling during HCV-induced HCC progression.

Extending our molecular findings into an immunological context, in silico immune infiltration analyses were performed to evaluate the associations between gene expression and immune cell composition in HCC. Our analyses showed strong positive correlations between these genes and multiple immune cell subsets, including macrophages, neutrophils, and both CD4⁺ and CD8⁺ T cells, indicating their involvement in orchestrating tumor–immune interactions. Importantly, these associations were derived from bulk transcriptomic data and therefore represent composite signals arising from both malignant hepatocytes and non-parenchymal immune populations. Consequently, the observed correlations may reflect differences in immune cell abundance, spatial distribution, or activation state, rather than direct transcriptional regulation within hepatocytes. Although prior studies have implicated SOX9, TNFAIP3, and FOSL2 in inflammatory and immune-related pathways, their precise mechanistic roles within the HCC immune microenvironment remain incompletely defined. These findings thus highlight biologically plausible interactions that warrant further functional investigation to determine whether these genes actively participate in shaping immune microenvironmental dynamics or primarily reflect broader immunologic remodeling within the tumor milieu.

Within this context, SOX9, acting downstream of IL-6/STAT3 and TGF-β signaling, has been implicated in EMT and chemokine-mediated immune recruitment [72, 73]. TNFAIP3 displayed the strongest correlations with immune-related genes in our analysis and was associated with immune checkpoint molecules such as CTLA4 and BTLA. This aligns with its established function in driving immunosuppressive macrophage polarization, especially in viral-related HCC [74]. FOSL2 correlated with IL2RA and IL6, supporting its role in macrophage activation and inflammatory tissue remodeling [38, 39]. Taken together, these results suggest that elevated expression of these genes contributes to an inflamed yet immunosuppressive tumor microenvironment that may facilitate HCC progression.

To address the challenges posed by invasive liver biopsies and tumor heterogeneity, PBMCs present a reproducible and minimally invasive source for evaluating systemic immune alterations and for exploring prognostic biomarkers relevant to HCC progression [25, 75, 76]. Employing PBMC-derived gene expression, our ROC analyses demonstrated the robust diagnostic accuracy of SOX9 and TNFAIP3 in discriminating between patient subgroups. SOX9 showed high diagnostic accuracy in post-SVR-HCC patients (AUC = 0.92, specificity 97.22%, *P* < 0.0001), while its performance was limited in DAA-naïve HCV-HCC patients (AUC = 0.5251). Likewise, TNFAIP3 exhibited strong diagnostic potential, particularly in post-SVR-HCC patients (AUC = 0.85) and CHC-no HCC (AUC = 0.84), with high specificity across groups (*P* < 0.0001). In contrast, FOSL2 displayed weak and mostly non-significant diagnostic performance in all groups. Overall, our findings underscore the diagnostic utility of SOX9 and TNFAIP3 as reliable biomarkers for HCC detection, particularly after DAA therapy and in chronic HCV, offering enhanced sensitivity and specificity over conventional markers. Although DAAs effectively eradicate HCV, they do not fully reverse virus-induced epigenetic and immune alterations. Persistent dysregulation of SOX9 and TNFAIP3 may sustain inflammatory signaling and immune infiltration, fostering an oncogenic microenvironment even after viral clearance. These findings highlight the need for post-DAA surveillance strategies that incorporate molecular and immune profiling, with SOX9 and TNFAIP3 serving as potential biomarkers for early HCC detection and personalized therapeutic intervention.

### Limitations

This study aimed to identify transcriptional signatures associated with HCC in the post-SVR setting by analyzing HCC tissues collectively across BCLC stages. However, HCC progression is accompanied by dynamic, large-scale transcriptomic reprogramming, as documented in prior studies [77], and pooling samples across BCLC stages makes it unclear whether the identified genes behave differently during disease progression. Larger, stage-stratified cohorts will be needed to determine whether this gene signature is consistent across HCC or exhibits stage-specific patterns. In addition, comparison of post-SVR patients with and without HCC would be best addressed in large longitudinal cohorts with extended follow-up, as residual HCC risk after viral cure is driven by stable molecular and epigenetic alterations rather than transient transcriptional changes [13, 53, 78, 79]. Given the limited follow-up in the present study, inclusion of a post-SVR/no-HCC group could result in misclassification of individuals who subsequently develop HCC. Instead, this study was designed as a foundational cross-sectional analysis to identify transcriptional signatures associated with established HCC in the post-SVR setting, providing a necessary framework for subsequent prognostic evaluation. Accordingly, the predictive value of the studied genes for future HCC development requires validation in longitudinal cohorts with extended follow-up. Finally, our cohort was composed exclusively of patients infected with HCV genotype 4, the predominant strain in Egypt and the Middle East and North Africa (MENA) region. This homogeneity minimizes inter-genotypic confounding and strengthens internal validity. While key oncogenic mechanisms are likely conserved, the generalizability of our gene-expression signature to other genotypes requires validation in diverse cohorts.

## Supplementary Information

Below is the link to the electronic supplementary material.

Supplementary Material 1

## Data Availability

The datasets generated and/or analyzed during the current study are not publicly available due to confidentiality of patient data but are available from the corresponding author on reasonable request.
