# Supplementary material for: SOX9 and TNFAIP3 dysregulation in HCV-associated HCC after DAA therapy: insights into post-viral oncogenic memory
Source: Infect Agent Cancer. 2026 Feb 19;21:26. doi: 10.1186/s13027-026-00735-w (PMC13020310; doi:10.1186/s13027-026-00735-w)
Supplement: Supplementary file 1 — Supplementary Material 1 [file 13027_2026_735_MOESM1_ESM.docx]

Supplementary data:

**Supplementary Table S1: The Relative Expression of SOX9, TNFA1P3, FOSL2 in PBMCs and Liver Tissues:-**

|  | **SOX 9** | **TNFAIP3** | **FOSL2** |
| --- | --- | --- | --- |
|  | **relative expression** | **relative expression** | **relative expression** |
|  | (mean±SD) | (mean±SD) | (mean±SD) |
|  |  | **Serum** |  |
| **Control** | 1.243 ± 1.052 | 1.056 ± 0.8884 | 1.265 ± 1.011 |
|  |  |  |  |
| **Post-SVR-HCC** | 14.48 ± 20.22 | 4.703 ± 6.834 | 0.9701 ± 0.7568 |
| **DAA-naïve HCV-HCC** | 2.757 ± 5.763 | 1.322 ± 1.533 | 0.9906 ± 0.8361 |
| **CHC-no HCC** | 13.31 ± 19.82 | 2.964 ± 2.304 | 1.579 ± 1.003 |
| **Non-HCV-HCC** | 0.6795 ± 0.681 | 5.282 ± 6.162 | 1.018 ± 1.142 |
| **P value** | 0.0001* | 0.0001* | 0.0161* |
| **Tissue** | | | |
| **Control** | 1.571 ± 2.887 | 1.063 ± 0.6386 | 1.047 ± 0.3766 |
| **Post-SVR-HCC** | 10.53 ± 6.431 | 3.372 ± 2.110 | 1.339 ± 0.8046 |
| **DAA-naïve HCV-HCC** | 9.485 ± 6.923 | 2.931 ± 1.618 | 1.631 ± 1.690 |
| **Non-HCV-HCC** | 5.337 ± 2.302 | 3.14 ± 2.709 | 1.618 ± 1.195 |
| **P value** | 0.0022** | 0.0472* | 0.8704 |

**Supplementary** Table S2**: Correlation between the expression of selected genes in HCC tissues and PBMCs**

|  | **Groups** | **r** | **CI 95%** | **P value** |
| --- | --- | --- | --- | --- |
| **SOX9** | Tissues Post-SVR-HCC vs  PBMCs Post-SVR-HCC | 0.9654 | 0.8853 to 0.9899 | <0.0001* |
|  | Tissues DAA-naïve HCV-HCC vs  PBMCs DAA-naïve HCV-HCC | 0.7514 | 0.5068 to 0.8841 | <0.0001 |
|  | Tissues non-HCV-HCC vs  PBMCs non-HCV-HCC | 0.8977 | 0.6452 to 0.9734 | 0.0002* |
| **TNFAIP3** | Tissues Post-SVR-HCC vs  PBMCs Post-SVR-HCC | 0.9779 | 0.8951 to 0.9955 | <0.0001 |
|  | Tissues DAA-naïve HCV-HCC vs  PBMCs DAA-naïve HCV-HCC | 0.9097 | 0.7327 to 0.9714 | <0.0001* |
|  | Tissues non-HCVHCC vs  PBMCs non-HCV-HCC | 0.9355 | 0.8039 to 0.9798 | <0.0001* |
| **FOSL2** | Tissues Post-SVR-HCC vs  PBMCs Post-SVR-HCC | 0.9823 | 0.9153 to 0.9964 | <0.0001* |
|  | Tissues DAA-naïve HCV-HCC vs  PBMCs DAA-naïve HCV-HCC | 0.7946 | 0.4333 to 0.9359 | <0.0012* |
|  | Tissues non-HCV-HCC vs  PBMCs non-HCV-HCC | 0.7333 | 0.3541 to 0.9055 | <0.0019* |

**Supplementary Table S3: Diagnostic capacity of the Studied Gene Expression:-**

|  | **AUC** | **Cut off** | **Sensitivity%** | **Specificity%** | **95% CI** | **P value** |
| --- | --- | --- | --- | --- | --- | --- |
| **SOX9** | | | | | | |
| **Post-SVR-HCC** | 0.9167 | 3.182 | 66.67% | 97.22% | 0.7344-0.8990 | <0.0001* |
| DAA-naïve HCV-HCC | 0.5251 | 1.211 | 50% | 58.33% | 0.3715-0.6788 | 0.7374 |
| **CHC-no HCC** | 0.8379 | 2.739 | 65.22% | 86.11% | 0.6159-0.9034 | 0.0008* |
| **Non-HCV-HCC** | 0.6706 | 0.7740 | 60% | 61.11% | 0.5386-0.8027 | 0.0199* |
| **TNFAIP3** | | | | | | |
| **Post-SVR-HCC** | 0.8488 | 1.366 | 68.57% | 76.92% | 0.7050-0.87727 | <0.0001* |
| **DAA-naïve HCV-HCC** | 0.5393 | 0.6930 | 55.17% | 56.41% | 0.3873-0.6914 | 0.5811 |
| **CHC-no HCC** | 0.8324 | 1.772 | 68.97% | 87.18% | 0.7292-0.9365 | <0.0001* |
| **Non-HCV-HCC** | 0.7966 | 1.803 | 58.62% | 81.74 | 0.6891-0.9042 | <0.0001* |
| **FOSL2** | | | | | | |
| **Post-SVR-HCC** | 0.5874 | 0.6030 | 42.25% | 71.43% | 0.4802-0.6945 | 0.1217 |
| **DAA-naïve HCV-HCC** | 0.5956 | 0.6630 | 55.17% | 66.67% | 0.4583-0.7330 | 0.1730 |
| **CHC-no HCC** | 0.6658 | > 1.381 | 50% | 66.67% | 0.5357 - 0.7958 | 0.0224* |
| **Non-HCV-HCC** | 0.5849 | 0.7605 | 43.33% | 57.14% | 0.4493-0.7205 | 0.2216 |
